# Postoperative Sore Throat After Laryngoscopy With Macintosh or Glide Scope Video Laryngoscope Blade in Normal Airway Patients

**DOI:** 10.5812/aapm.15136

**Published:** 2014-02-16

**Authors:** Atabak Najafi, Farsad Imani, Jalil Makarem, Mohammad Reza Khajavi, Farhad Etezadi, Shirin Habibi, Reza Shariat Moharari

**Affiliations:** 1 Department of Anesthesiology, Sina Hospital, Tehran University of Medical Sciences, Tehran, Iran; 2 Department of Anesthesiology, Imam Khomeini Medical Center, Tehran University of Medical Sciences, Tehran, Iran

**Keywords:** Hoarseness, Intubation, Laryngoscopy, Pharyngitis

## Abstract

**Background:**

The Glide Scope videolaryngoscope provides a suitable view for intubation, with less force required.

**Objectives:**

The present study was conducted, to compare postoperative sore throat and hoarseness after laryngoscopy and intubation, by Macintosh blade or Glide Scope video laryngoscope in normal airway patients.

**Patients and Methods:**

Three hundred patients were randomly allocated into two groups of 150: Macintosh blade laryngoscope or Glide Scope video laryngoscope. The patients were evaluated for 48 hours for sore throat and hoarseness by an interview.

**Results:**

The incidence and severity of sore throat in the Glide Scope group, at 6, 24 and 48 hours after the operation, were significantly lower than in the Macintosh laryngoscope group. In addition, the incidence of hoarseness in the Glide Scope group, at 6 and 24 hours after the operation, were significantly lower than in the Macintosh laryngoscope group. The incidence and severity of sore throat in men, at 6 and 24 hours after the operation, were significantly lower than in the women.

**Conclusions:**

The incidence and severity of sore throat and hoarseness after tracheal intubation by Glide Scope were lower than in the Macintosh laryngoscope. The incidence and severity of sore throat were increased by intubation and longer operation times.

## 1. Background

Direct laryngoscopy guided by a Macintosh curved blade is the standard, traditional method of endotracheal intubation in patients under general anesthesia (1-3). In this method, significant force is usually applied in order to provide a good laryngoscopic view for intubation. Hemodynamic adverse events, soft tissue damage, and postoperative sore throat, with a reported incidence of up to 90%, are frequent problems after intubation (4-11). Some factors are noted to be effective in decreasing the frequency of postoperative cough and sore throat, e.g. using lidocaine to inflate the endotracheal tube (ETT) cuff, or IV lidocaine at the end of surgery (12), but some factors, e.g. curved or straight laryngoscope blades (4), have been shown to be ineffective.

Although another indication of the Glide Scope has been applied (13), it has been used with increasing frequency for tracheal intubation (14). The Glide Scope videolaryngoscope provides a suitable view for intubation with less force needed (15).

## 2. Objectives

The present study was conducted to compare postoperative sore throat and hoarseness, after laryngoscopy and intubation by Macintosh blade or GSL, in normal airway patients.

## 3. Patients and Methods

The study protocol was approved by the Ethics Committee of Tehran University of Medical Sciences. The study was explained to all patients, and informed consent was obtained. In this randomized double blinded clinical trial, all of the patients were; ASA physical status I and II, with MET > 4, who were scheduled for elective surgery under general anesthesia in the supine position, from December 2012 till May 2013. Exclusion criteria were: age < 18 years or age > 60 years; any anatomical abnormality in the head, neck or face; any ENT, neck or thoracic surgery; smoking history; edentulous patients; estimated surgery time > 4 hours; any clinical evidence of active pulmonary disease; common cold during the recent two weeks; limited mouth opening or neck extension.

Three hundred patients were enrolled in the study. They were randomly allocated into two groups of 150 by the block randomization method: Macintosh blade laryngoscopy (ML) or Glide Scope videolaryngoscope (GVL). In the ML group; size 3 (for women) and size 4 (for men) Macintosh blades were used, and in the GVL group; size 4 reusable blades were applied in all cases. Patients and the anesthesia resident, who evaluated the patients postoperatively, were blinded. Patients were evaluated for cough and bucking at the time of extubation. After finishing the operation at 1, 6, 24 and 48 hours, the patients were visited by an anesthetist, who was blinded to the way of intubation, and they were asked about a sore throat and its severity on a three step scale (no pain, low pain and high pain) and hoarseness, (via an interview) too.

The patients were pre-oxygenated for 2 minutes with 100% O2. The patients were premedicated with; midazolam 0.03 µg/ kg, and fentanyl 3 µg/ kg. Induction of the anesthesia was performed by thiopental Na 5mg/kg, and neuromuscular paralysis was facilitated by atracurium 0.5 mg/kg. All patients were orally intubated after 3 minutes of induction by one anesthesiologist in both groups; ML or GVL groups. Intubation was performed using a low pressure cuff (Supa Inc., Tehran, Iran) with an inner diameter of 7.5 and 8 mm in women and men, respectively. No gel or lidocaine spray was used. The cuff was inflated at pressure to approximately 20-25 cmH20. Anesthesia was maintained by isoflurane 1.5-2%, and repeated doses of fentanyl and atracurium, as needed. Thirty minutes before extubation, all patients received fentanyl 50 µg intravenously.

More than three tries in the ML and GVL groups were considered to be a failure of laryngoscopy, and alternative methods (in the ML group, Glide Scope videolaryngoscope and in the GVL group, a Macintosh blade was used for intubation, and fiberoptic guided intubation was considered if required) were applied. 

Age, sex, weight, ASA classification, anesthesia duration (induction till discontinuation of maintenance anesthesia), extubation time (discontinuation of maintenance anesthesia till extubation), existence of postoperative sore throat, hoarseness, and dysphagia, were measured in the patients.

A Kolmogorov-Smirnov test for goodness of fit was performed. The continuous variables were presented as mean ± SD. A Student's T test was used for comparison of means between the groups. Chi square tests were used for the categorical variables. A P value < 0.05 was considered to be significant.

## 4. Results

The two groups were comparable with respect to; age, sex, ASA class, and duration of operation (Table 1). 

**Table 1.** Basic Variables in the Two Groups

	GVL ^a^ Group	ML ^a^ Group	P value
**Age, Mean ± SD, y**	39.1 ± 7.6	40.2 ± 7.2	0.44
**Sex, Male, No. (%)**	67 (44.7)	70 (46.7)	0.72
**ASA Class, No.**			
I	125	127	0.75
II	25	23	
**Mallampati, No. (%)**			
I	71 (47)	85 (56.7)	
II	48 (32)	40 (26)	0.36
III	18 (12)	17 (11.3)	
**IV**	13 (8.7)	8 (5.3)	
**Duration of operation, Mean **±**SD, Min**	133.6 ± 23.2	127.7 ± 21.1	0.23

^a^ Abbreviations: GVL, Glide Scope videolaryngoscope; ML, Macintosh blade laryngoscopy.

The mean ± SD duration of intubation in the GVL and ML groups was; 37.2 ± 6.4 and 25.6±4.1 seconds, respectively P < 0.001). The mean ± SD extubation time in the GVL group was 14.9±2.4 minutes and in the ML group 12.5 ± 2.1 minutes (P < 0.001). The incidence of coughing or bucking in the GVL and ML groups were; 21patients (14%), and 25 patients (16.7%), respectively (P = 0.52).

The incidence and severity of sore throat (low & high pain), in the ML group at 6, 24 and 48 hours after the operation, were significantly higher than in the GVL group (Table 2). The incidence of hoarseness in the GVL group at 6 and 24 hours after the operation, were significantly lower than in the ML group. The incidence and severity of sore throat, in men at 6 and 24 hours after the operation, were significantly lower than in the women (P < 0.001). The incidence and severity of a sore throat were increased by intubation and increasedoperation time.

**Table 2.** Incidence of Postoperative Sore Throat, Hoarseness, and Dysphagia, in the Two Groups

	GVL ^a^ Group, No. (%)	ML ^a^ Group, No. (%)	P value
Sore Throat			
1 h	29 (19.3)	42 (28)	0.08
6 h	42 (28)	81 (54)	< 0.001
24 h	34 (22.7)	81 (54)	< 0.001
48 h	28 (18.7)	49 (32.7)	0.006
**Hoarseness**			
1 h	29 (19.3)	43 (28.7)	0.06
6 h	37 (24.6)	70 (46.7)	< 0.001
24 h	30 (20)	64 (42.7)	< 0.001
48 h	24 (16)	32 (21)	0.23
**Dysphagia**			
1 h	17 (11.3)	22 (14.7)	0.39
6 h	28 (18.7)	37 (24.7)	0.21
24 h	20 (13)	29 (19.3)	0.16
48 h	8 (5.3)	10 (6.7)	0.63

^a^ Abbreviations: GVL, Glide Scope videolaryngoscope; ML, Macintosh blade laryngoscopy.

## 5. Discussion

Postoperative sore throat and hoarseness are frequent problems after a general anesthesia. However, these complications are not major or life threatening. Considering their pathophysiological mechanisms and risk factors will help us to mitigate the incidence and severity of the problem. Pharyngotracheal tissue damage, due to laryngoscopy and intubation, are the main mechanisms. Laryngoscopy and endotracheal tube specificities, use of lubricants for intubation, emergency settings, anatomy of the airway, and difficult intubation, are associated with postoperative sore throat and hoarseness (7, 16-23).

In this study we evaluated the effect of the laryngoscopy method, while other factors were controlled. According to our findings, postoperative sore throat and hoarseness were less frequent in the GVL group than in the ML group. GVL facilitates the visualization of the glottic inlet (24). The 60° curvature of the Glide Scope blade with a light source and digital camera at the tip of it, enables intubation without the need to align the oral, pharyngeal and tracheal axes (25). It also needs less forceful laryngoscopy and results in less tissue trauma (15). It is rational to attribute less postoperative sore throat and hoarseness to less tissue trauma in the GVL group. A similar finding has been reported in a limited number of reports (26-28). The Glide Scope facilitated nasotracheal intubation to a greater degree than the Macintosh laryngoscope in adults with normal airways, which produced a lower incidence of sore throat. 

Other GVL related complications, such as; soft palate tearing (29), mucosal bleeding, and lip bleeding (28), have been reported, but in our study none of these problems occurred. The main limitation of the GVL is the difficulty in advancing the tracheal tube, because it requires sufficient hand-eye coordination, and in most cases blind passage of a tracheal tube from the mouth to the larynx while the operator only observes the display screen, and this was associated with some injuries. The length of time of a laryngoscopy by GVL was longer than ML in this study, as reported previously (30). However, in a recently published systematic review, a significant heterogeneity in the time of intubation by GVL in comparison with a ML was reported (24). As a result, a clear conclusion about the estimated time of intubation with the guidance of a Glide Scope is not possible. 

As was previously showed in a manikin CPR scenario, the application of a Glide Scope was associated with higher intubation success rates in medical practitioners inexperienced in intubation (31). Incorrect esophageal intubation was significantly reduced by GVL (32). In difficult airway patients in emergency settings, the GVL had a higher success rate at first attempt than the ML (33). As sore throat and hoarseness seem to be less by Glide Scope than ML, future studies should consider whether it would be safe to intubate with less depth of anesthesia (by guide of BIS). As the degree of muscle relaxation before laryngoscopy (validated with objective assessment), endotracheal tube cuff pressure, application of NGT/OGT intra operatively, and application of cricoid pressure, influence the rate of postoperative sore throat and hoarseness (34), thus we suggest that these variables should also be considered in future studies.

In conclusion, intubation by GVL was associated with less postoperative sore throat and hoarseness in normal airway patients. The failure rate of intubation was decreased significantly by Glide Scope intubation. We recommend GVL for all intubations, even by trained anesthesiologists, in order to decrease failure and complication rates of intubation.
